# Speech outcomes after palatal closure in 3–7-year-old children

**DOI:** 10.1016/j.bjorl.2020.08.005

**Published:** 2020-09-30

**Authors:** Parisa Rezaei, Marziyeh Poorjavad, Hossein Abdali

**Affiliations:** a Isfahan University of Medical Sciences, Craniofacial and Cleft Research Center, Isfahan, Iran; b Isfahan University of Medical Sciences, School of Rehabilitation Sciences, Department of Speech Therapy, Isfahan, Iran; c Isfahan University of Medical Sciences, Department of Plastic Surgery, Isfahan, Iran

**Keywords:** Speech disorders, Cleft palate, Child, Velopharyngeal insufficiency, Surgery

## Abstract

**Introduction:**

One of the main goals of the team approach in management of oro-facial clefts is to help the children with cleft palate have adequate speech development.

**Objective:**

The present study aimed to investigate the prevalence of articulation and resonance disorders following palate closure in children who were visited for routine examination by the Isfahan Cleft Care Team between 2011 and 2015, and to study the impact of cleft type and age at the time of palatoplasty on speech outcomes.

**Methods:**

Clinical records of 180 preschool children with repaired cleft palate were reviewed. The percentage of children demonstrating hypernasality, nasal emission, nasal turbulence, and compensatory misarticulations was calculated. The relationship between cleft type and age at the time of palatal surgery, as independent variables, and speech outcomes were examined.

**Results:**

67.7 and 64.5 percent of the children demonstrated respectively moderate/severe hypernasality and nasal emission, and 71.1 percent produced compensatory misarticulations. Age at the time of palatal repair was significantly associated with compensatory misarticulations and also with moderate/severe hypernasality. The prevalence of compensatory misarticulations, significant hypernasality, nasal emission and also nasal turbulence was not significantly different in various types of cleft.

**Conclusions:**

We observed a high prevalence of different speech disorders in preschool children with repaired cleft palate compared to other studies. This can be partly due to late palatal repair in the studied population. Despite many advances in cleft palate management programs in Iran, there are still many children who do not access the interdisciplinary team cares in their early childhood. We should, therefore, try to increase accessibility of appropriate and timely management services to all Iranian children with cleft lip/palate.

## Introduction

Velopharyngeal insufficiency (VPI) following primary palatal closure can result in excessive nasal resonance or hypernasality in patients with cleft palate. In addition to resonance disorders, VPI can adversely affect phonetic and phonological development.^1^ Nasal air emission, nasal grimace,^2^ consonant deletion, weak articulation of high pressure consonants, compensatory articulation errors (compensatory misarticulation–CMA) are prevalent phonetic errors in children with cleft palate. One of the main goals of team management is to help the children with cleft palate have adequate speech development.^3^

There is empirical evidence suggesting that “team management program” can affect the prevalence of complications of cleft palate, especially speech outcomes.^4^, ^5^ The surgical strategies, the surgeon’s experience,^2^ the timing of surgeries, the efficacy of early consultations and intervention programs^5^ regarding ear and hearing problems and also regarding speech and language development, the extent of provided psychosocial supports to the parents, and the timing and quality of provided speech/language/hearing interventions are factors that seem to be able to dramatically alter the speech/language outcomes in cleft palate children. Chapman and et al.^1^ indicated that chronological age and lexical status at the time of primary palatal surgery can significantly change the speech outcome of children with cleft palate. They found that 43% of children with non-syndromic cleft palate were enrolled in speech intervention program at 3 years of age. Furthermore 43% of these children showed significant hypernasality. However, children of a younger age and less advanced lexicon at the time of palatal surgery had better speech/articulation and resonance outcomes.^1^ Hardin-Jones and Jones^5^ reported that 37% and 25% of preschoolers (mean age 42 months) with repaired cleft palate showed respectively moderate- severe hypernasality and compensatory articulation error, respectively. Their analysis revealed that there are significant relationships between age at palatal surgery and cleft type, as independent variables, and the prevalence of moderate- severe hypernasality. The authors underlined, therefore, the need for early palatal repair (before than 13 months of age).^5^ Paliobei V, Psifidis A, Anagnostopoulos D^6^ evaluated resonance and articulation abilities of cleft palate patients who had received a delayed hard palate closure between 18 and 24 months of age. The authors found that 40.5% and 28.5% of their patients continue to demonstrate hypernasality and CMA between 5 and 15 years of age. They also showed that hypernasality is more frequent in patients with cleft palate (hard and soft palate–CP) compared to those with unilateral/bilateral cleft lip and palate (UCLP/BCLP).^6^ Ruiter et al.^7^ reported hypernasality and articulation problems, respectively, in 38 and 57 percent of children with cleft lip and/or palate at 20–46 months of age. Following a multidisciplinary approach to treatment, however, the prevalence of these problems decreased to respectively 10% and 25% at 54–76 months of age. Their results also showed that children with BCLP have the most speech problems compared with children with UCLP and CP.^7^

In addition to cleft type and factors associated with management protocol, Albustanji et al.^8^ suggested that the phonology of studied language should also be considered when compensatory articulations are assessed in cleft palate children. Despite early palatal closure in their study (mean at 6.7 months), 74% of Saudi children with cleft lip/palate showed speech abnormalities (71% articulation errors; 64% hypernasality) at 6–15 years of age (mean age 6.7 years). They concluded that the use of pharyngeal fricatives and glottal stops is frequent in Arabic-speaking children with repaired CLP because they are phonemic in Arabic phonological repertoire.^8^

Compared with studies^5^, ^6^, ^7^ performed in other countries, previous studies^9^, ^10^ in our clinic, the Isfahan Cleft Care Team (ICCT), reported a higher prevalence of hypernasality and compensatory errors among children with repaired cleft palate. Considering the high reported prevalence of speech abnormalities in preschoolers with repaired cleft palate who were assessed in our clinic, this study aimed to investigate the prevalence and types of articulation and resonance disorders among all 3–7 year old CLP children who were assessed in this clinic between 2011 and 2015. The findings will help to identify the strengths and weaknesses of provided services. The impact of cleft type and age at the time of palatal surgery on speech outcomes was also investigated.

## Methods

The study was approved by the Medical Research Ethics Committee of Isfahan University of Medical Sciences (IR. MUI.MED.REC.1398.418). Clinical records of all patients with different types of clefts, who were visited in ICCT between the years 2011–2015, were reviewed. The Isfahan Cleft Care Team (ICCT) at Isfahan University of Medical Sciences provides the comprehensive and interdisciplinary care of patients with cleft lip/palate since 2005 for the first time in Iran. In ICCT, patients with cleft palate are assessed by a team consisted of speech and language pathologists (SLPs), pediatric/plastic surgeon, otorhinolaryngologist, dentist and orthodontist, maxillofacial surgeon, geneticist, and nurse. SLPs usually evaluate patients' speech individually one day before the team session and make the appropriate referrals for instrumental examinations. They also manage the session and report patients’ condition for all team members. A pediatric/plastic surgeon evaluates palate structure and function and investigates the surgery choices. The otorhinolaryngologist examines patients’ ear and, if needed, refers them to audiologist. He also performs the nasoendoscopic evaluation. Patients are examined for dental and occlusal abnormalities by a dentist and orthodontist. Patients’ jaws and alveolar ridges are examined by the maxillofacial surgeon. A geneticist evaluates patients for probable genetic syndromes and also counsels parents. A nurse provides guidance regarding feeding problems in infants with cleft palate. In the team session, the patients’ treatment priorities are determined based on the results of all performed evaluations.

Since patients had received primary palatal repair at different centers across the country, different surgical techniques had been used. However, most of the primary surgeries were done using either the two-flap or push-back techniques. Sommerlad Intravelarveloplasty (SIVV) has also been used in a small number of patients since 2011. Patients were included if they were 3–7 years old at the time of the first speech assessment in our clinic, if Persian was their first language, and if they didn’t suffer from sensory- neural hearing loss,^1^, ^11^ identified cognitive disorders or congenital syndromes. The patients who had received the secondary surgery at the time of the first speech evaluation in our clinic or who suffer from non-cleft VPI or lip cleft only were excluded from the study. Speech evaluations in our cleft palate clinic are performed by two veteran experienced speech and language pathologists (SLP) and were being reported based on the Universal Parameters guidelines^12^ until 2016. The patients were asked to repeat the Persian words and sentences^13^ with high-pressure consonants developed according to these guidelines.^12^ Intra- and inter-rater reliability of this “cleft palate speech assessment test-Farsi version” has been reported respectively 0.97 and 0.95.^13^ The patient file was excluded if the speech assessment was not performed based on universal parameters guidelines or if syllable repetition tasks were used for speech sampling due to the child’s language delay or his/her non-cooperative behavior. In addition, those files in which type of cleft or age of primary palatoplasty were not recorded and those with two different ages for hard and soft palate surgery (two-stage surgeries) were excluded.

According to the Universal Parameters for reporting speech outcomes,^12^ hypernasality were assessed using a four-point scale (0 = Within normal limits (WNL); 1 = Mild hypernasality; 2 = Moderate hypernasality; 3 = Severe hypernasality). Ratings for nasal emission (NE) and also nasal turbulence (NT) were made using a binary system in which 0 = WNL/none and 1 = Present. Compensatory errors were recorded during repetition of the Persian words and sentences.^13^ According to the Peterson-Falzone et al. description,^14^ compensatory errors were categorized into six types: glottal stop, pharyngeal stop, pharyngeal fricative, pharyngeal affricative, posterior nasal fricative, and mid dorsum palatal stop/fricative.

Children were assigned to two groups according to their age at palatal surgery; the group of early surgery consisted of children who had surgery before 13 months of age and the group of late surgery included of those having surgery after 13 months of age. The percentage of children demonstrating each of the following characteristics was reported in each group and also in total: hypernasality, nasal emission, nasal turbulence, and compensatory errors. Chi-square tests were performed to compare the prevalence of these characteristics between two groups. The relationships between history of speech therapy and the studied speech problems were also explored by Chi-square tests. An independent sample *t* test was conducted to compare mean of age at the time of palatal surgery in children with and without CMA and also in children with and without moderate-severe hypernasality, nasal emission and nasal turbulence. In order to investigate the relationship between cleft type and speech disorders, we divided the patients into 3 groups. Group I included the patients with clefts involving both the hard and soft palate (i.e. bilateral cleft lip and palate [BCLP], unilateral cleft lip and palate [UCLP] and cleft palate [CP]). Group II consisted of the patients with cleft of the soft palate only (SPO) and group III included the patients with submucous cleft palate (Sub M.C). Relationship between the cleft type and these speech problems was also examined using Chi-Square analysis. Data analyses were performed using SPSS statistical software (version 19.0; SPSS Inc., Chicago, IL) and statistical significance was set at *p* ≤ 0.05.

## Results

**Figure 1:**
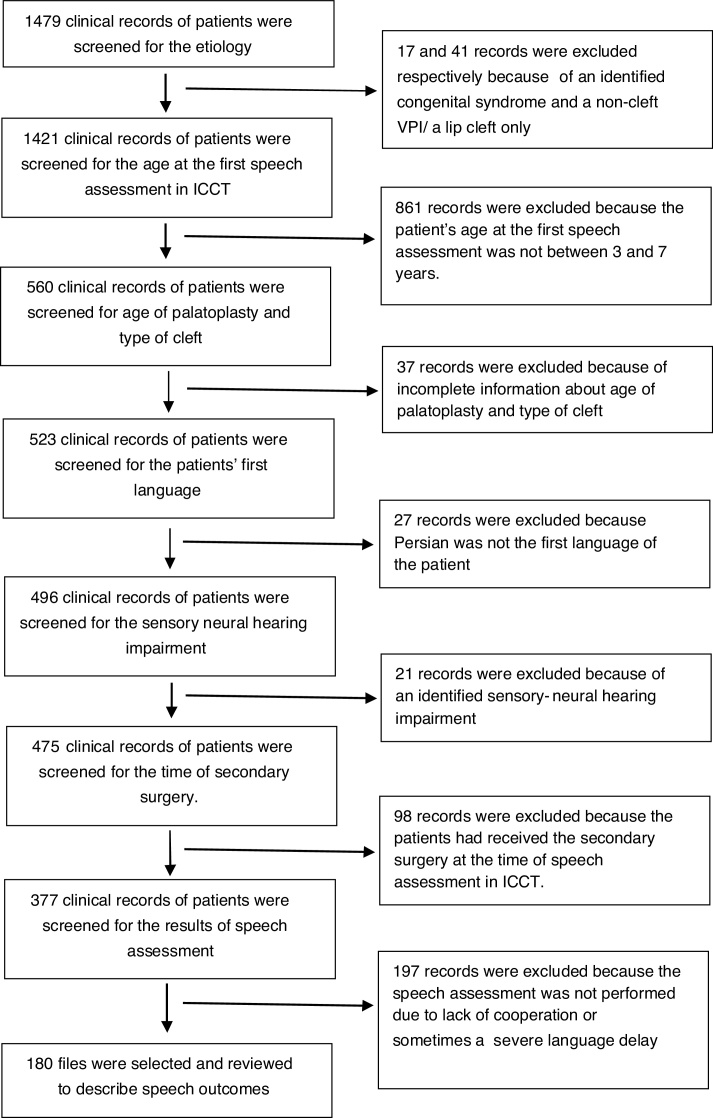
Patient inclusion and exclusion algorithm.

A total of 1479 clinical records of patients who were assessed in ICCT between 2011 and 2015 were screened. After excluding 1299 records, 180 files were finally selected and reviewed to describe speech outcomes. Fig. 1 illustrates the inclusion and exclusion process. This population included 102 boys (56.7%) and 78 girls (43.3%). The mean of age at palatal surgery and speech assessment was 16.7 ± 11.97 months (range: 5 to 72 months) and 53.47 ± 15.18 months (range: 36 to 84 months), respectively. The average interval between palatal surgery and speech evaluation was 36.78 ± 16.82 months (range from 3 months to 75 months). Ninety patients underwent palatal repair before 13 months of age (the early surgery group) and 90 patients had surgery at the age of 13 months or older (the late surgery group). Sixty-eight patients (37.8%) had a history of middle ear diseases like otitis media. At the time of speech evaluation, 27 children (15%) showed conductive hearing loss (hearing threshold >15 dB) and 97 patients (53.9%) had normal hearing. The results of hearing tests were not recorded in 56 files (31.1%). Seventy-eight children (43.3%) had a history of nasal regurgitation. Only 69 patients (38.3%) had received speech therapy services before coming to our center. The detailed information regarding received services (including type, goals and frequency of therapy, and its general results) was not recorded in the files; 20.6, 34.4 and 18.3 percent of children suffered respectively from BCLP, UCLP and CP (group I = 73.3%). SPO and Sub M.C were observed respectively in, 20 and 6.7 percent of the studied children (groups II and III).

In total 128 (71.1%) children had produced CMA and 111 (64.5%) and 13 (7.2%) children respectively had demonstrated nasal emission and nasal turbulence in their speech. Moderate-severe hypernasality had been recorded in speech of 113 children (67.7%). Table 1 shows the percentages of children who produced CMA, or demonstrated degrees of hypernasality, nasal emission, and nasal turbulence in two groups based on the age of palatal closure. There was a significant higher percentage of children producing CMA in the late surgery group.

**Table 1:** Percentage of children with speech abnormalities associated with age at surgery.

	Early surgery group	Late surgery group	*p*	χ^2^
CMA	57 (63.3%)	71 (78.9%)	0.021^a^	5.30
Nasal emission^b^	54 (61.4%)	57 (67.9%)	0.37	0.79
Nasal turbulence	4 (4.4%)	9 (10%)	0.15	2.07
Hypernasality^c^	71 (82.6%)	69 (85.2%)	0.086^d^	2.95
	Mild: 18 (20.9%)	Mild: 9 (11.1%)		
	Moderate: 17 (19.7%)	Moderate: 17 (21%)		
	Severe: 36 (41.9%)	Severe: 43 (53.1%)		

a Significant at *p* = 0.05.

b Nasal emission had not been assessed in 8 children due to lack of voiceless high-pressure oral consonants. So, the prevalence was calculated based on 172 cases; 88 patients in early surgery group and 84 patients in late surgery group.

c Hypernasality had not been assessed in 13 children due to lack of voiced high-pressure oral consonants. So, the prevalence was calculated based on 167 cases; 86 patients in early surgery group and 81 patients in late surgery group.

d The comparison was made between percentage of children who demonstrated moderate or severe hypernasality in each group.

The prevalence of different types of CMA is reported in Table 2. Glottal stops were the most prevalent type of CMA in Persian-speaking children with cleft palate after primary palatoplasty. While only 43 of 128 children (33.6%) who produced CMA had a history of speech therapy, 50% of children without CMA (26 of 52 children) had received speech therapy services (*p* = 0.04, χ^2^ = 4.21). Chi-square tests, however, did not demonstrate significant differences regarding history of speech therapy between children with and without NE, NT, and moderate- severe hypernasality (respectively *p* = 0.32, χ^2^ = 1.01, *p* = 0.56, χ^2^ = 0.34, *p* = 0.25, χ^2^ = 1.32).

**Table 2:** Percentage of children who produced different types of CMA.

Compensatory Misarticulation (CMA)	n	Percent^a^
Glottal stop (g.s)	78	44.57
Pharyngeal fricative (ph.fri)	38	21.71
Middorsum palatal stop	47	26.85
Pharyngeal affricative (ph.aff)	–	–
Posterior nasal fricative (p.n.f)	32	18.28
Pharyngeal stop (ph.s)	10	5.71

a The percentages were calculated based on n = 175. The type of CMA was not recorded in 5 files.

Table 3 indicates the mean age of children at the time of palatal surgery. Children who produced CMA compared to children without CMA had received palatal surgery at older ages. The age at palatal surgery was also associated with significant hypernasality. Children who demonstrated normal resonance or mild hypernasality had received palatal surgery at younger ages compared to children with moderate or severe hypernasality. Regarding nasal emission and nasal turbulence, although the mean age at palatal surgery was higher in children who demonstrated these problems compared to others, the differences were not statistically significant.

**Table 3:** Means and Standard Deviations (SD), *t* Values (*t*) and Significance Levels (p) for age of children at the time of palatal surgery.

Speech abnormalities		Mean ± SD (months)	*p*	*t*
CMA	Children with CMA	17.9 ± 12.6	0.028^a^	−2.22
	Children without CMA	13.9 ± 10.1		
Hypernasality	Children with moderate-severe hypernasality	17.6 ± 12.9	0.030^a^	−2.20
	Children without moderate-severe hypernasality	13.9 ± 8.6		
Nasal emission	Children with nasal emission	17.5 ± 12.9	0.11	−1.61
	Children without nasal emission	14.7 ± 9.6		
Nasal turbulence	Children with nasal turbulence	21.6 ± 16.1	0.13	−1.53
	Children without nasal turbulence	16.3 ± 11.6		

a Significant at *p* = 0.05.

Based on Chi- square analyses, no significant relationship was observed between cleft type and the number of children who produced CMA, as well as the number of those with moderate- severe hypernasality and also those with nasal emission or nasal turbulence. CMA, significant hypernasality and nasal emission had the least prevalence in children with cleft of the Soft Palate Only (SPO) (Table 4).

**Table 4:** Percentage of children with speech abnormalities by type of cleft.

	Group I (BCLP + UCLP + CP)	Group II (SPO)	Group III (Sub M.C)	*p*	χ^2^
	n/N (%)	n/N (%)	n/N (%)		
CMA	96/132 (72.7)	23/36 (63.9)	9/12 (75)	0.56	1.17
Moderate-severe hypernasality	87/125 (69.6)	18/31 (58.1)	8/11 (72.7)	0.44	1.65
Nasal emission	83/128 (64.8)	20/33 (60.6)	8/11 (72.7)	0.76	0.55
Nasal turbulence	8/132 (6)	4/36(11)	1/12(8.3)	0.58	1.11

## Discussion

The purpose of this study was to investigate and describe speech status of preschool children with repaired cleft palate who appeared for routine examination in ICCT from 2011 to 2015. We also investigated whether age at palatal surgery and cleft type may affect the speech status of cleft palate children at preschool ages. A relatively large number of children with repaired cleft palate were studied.

Our findings showed that velopharyngeal closure is inadequate in approximately two-thirds of children with repaired cleft palate who attend ICCT between 2011 and 2015 and they may need a secondary surgery.^1^ This percentage of significant hypernasality (67.7%) is much higher than those reported in other studies performed at other cleft palate centers (about 40%).^1^, ^5^, ^6^ In contrast, this finding is in line with those of Derakhshandeh and Poorjavad,^9^ Rezaei et al.,^10^ the earlier studies that reported the prevalence of moderate-severe hypernasality among preschool cleft palate children visited in ICCT respectively during 2005–2007, and 2006–2009.

Our findings also showed a higher rate of compensatory misarticulation (71.1%) compared with other studies (about 25%).^5^, ^6^, ^7^ These observations are again in line with the previous studies performed in ICCT.^9^, ^10^ The results indicated that only about one third of the studied children (38.3%) had received speech therapy services before assessing their speech in our clinic. It may indicate that, in our community, the majority of families with cleft palate children had neglected their child's speech sounds. It may also show that many families did not access speech therapy services or they were not appropriately referred to speech therapists before coming to our clinic. The findings also showed that a significantly higher percentage of children who did not produce CMA had received speech therapy services compared with children producing CMA (50% vs. 33.6%). Although the detailed information concerning these services was not available, we may conclude that they had been effective in reducing the compensatory errors in our children. Since nasal emission, nasal turbulence and hypernasality were not usually affected by behavioral therapies, it’s not surprising that there were not significant differences regarding history of speech therapy between children with and without these problems.

On the whole, our findings suggest that, between the years 2011–2015, an appreciable majority of Iranian children with repaired cleft palate continue to demonstrate significant speech problems after primary palatoplasty. Moreover, the language development of children had not been assessed in this study. Recently, Særvold et al.^15^ reported a significant association between the presence of hypernasality and lower language skills in 10-year-old children with cleft palate. They also found that children with reduced intelligibility have weaker reading comprehension skills. Their study introduced hypernasality and reduced intelligibility as clinical indicators of delay in language and reading skills in school-aged children with cleft palate.^15^ Accordingly in addition to speech defects, our studied children may also be at a high risk for language and reading deficits. However, it is worth mentioning that not all of these children received team management services (including primary surgery and early consultations and interventions regarding speech development) in ICCT. Many of them had received the primary surgical management and other services at the other treatment centers. They had come from across the country looking for an exact and comprehensive evaluation. Therefore, the results of the present study cannot evaluate the effects of interdisciplinary team care on the long-term speech outcomes of Iranian children with cleft palate. It should also be considered that those patients suffering from more obvious disorders may be more likely to seek evaluation and medical cares. The observed high prevalence of the history of middle ear diseases and nasal regurgitation in the studied patients also indicated that many of our patients had more complicated situations. We have, therefore, observed more patients with severe speech disorders in our studied population.

About half of the studied children produced glottal stops as substitutions for at least one sound. In agreement with Hardin-Jones and Jones,^5^ glottal stop was the most prevalent compensatory patterns in the cleft palate speech. None of children produced pharyngeal affricative. Hardin-Jones and Jones^5^ also reported only one patient producing pharyngeal affricate in their study. It seems that ease of the manner of articulation encourages many of children with cleft palate to produce glottal stops. In contrast, because of complexity of the manner of articulation, the less patients tend to produce pharyngeal affricates in their speech, especially in Persian in which words consisting affricates are not very frequent.^16^

The mean age at palatal surgery was higher in patients with different studied speech problems compared with those without these deficits. This relationship was significant regarding moderate-severe hypernasality and also regarding CMA. In agreement with Hardin-Jones and Jones^5^ and also Chapman et al.,^1^ the mean age of palatal surgery was significantly higher among children who demonstrated moderate-severe hypernasality. For normal motor speech development,^17^ velopharyngeal movements need to be integrated with other oral movements. A “critical sensitive period” at 4–6 months of age has been suggested for the sensorimotor integration of speech movements. The early palatal repair seems essential for efficient movement integration during child development. Further, children who produced CMA had been received the palatal surgery at the older ages compared to those without CMA. An early-repaired mechanism provides more opportunities for adequate phonological development. CMA as atypical patterns of articulation, therefore, are less likely to stabilize in the speech of child who is younger at the time of palatal surgery.^1^, ^5^ Given that children with cleft palate may begin to use the atypical articulatory patterns from prelinguistic period and in their early babbling,^18^ Chapman et al.^1^ suggested studying speech outcomes in children who received primary palatoplasty even earlier (by 6 months of age). In 180 of our studied children, however, only 4 (0.02%) were younger than 6 months at the time of their primary palatal repair. Thus we could not study the speech outcomes of very early palatal repair.

In our study, the mean age of palatal surgery in children without significant hypernasality and also in those without CMA was approximately 13 months. In order to have better speech outcomes, Hardin-Jones and Jones^5^ also suggested that primary palatal repair should not be performed later than 13 months of age. Our results showed that the mean age of palatal surgery in our total population was 16.7 ± 11.97 months and exactly fifty percent of the children had received the palatal surgery at the age of 13 months or older. Thus, the observed high prevalence of speech disorders in the studied children is partly due to late palatal repair. It is, however, worth considering that the age of children at the time of palatoplasty may also be associated to some extent with parenting style. In our experience, parents who had postponed their baby’s palatal repair based on non-medical reasons are less likely to participate in early intervention programs or to register their children for speech therapy. Therefore, their children are more likely to have weaker outcomes. Furthermore, our findings indicated that compared with other studies, the prevalence of different speech problems was also high among those children who received palatal surgery before 13 months. As mentioned above, therefore, the age at palatal surgery cannot be the only reason for the observed high prevalence of complications in our cleft palate children. Other studies need to be carried out to evaluate some other factors affecting the speech outcomes in our patients. Recently Mapar et al.^19^ studied a small population of patients with cleft palate who had received palatal repair using SIVV in ICCT between the years of 2011 and 2014 (n = 40). All the surgeries were performed by the ICCT’s surgeons experienced in SIVV technique between 3–5 years. They reported moderate and severe hypernasality only in 29.4% of patients who received the surgery before 12 months of age. In comparison with our results in patients who had palatal surgery before 13 months, this dramatic decrease in frequency of patients with significant hypernasality is encouraging for using this technique in children with cleft palate in ICCT.

There was not a significant relationship between cleft type and the prevalence of hypernasality, nasal emission, nasal turbulence or CMA. The percentage of children with speech problems was high and similar in all types of cleft. Hardin-Jones and Jones^5^ reported that significantly fewer children in the SPO group demonstrated moderate-severe hypernasality. They concluded that the severity of cleft can significantly affect speech outcomes.^5^ Our findings also showed that the studied speech disorders (except nasal turbulence) were less common in this group compared to the other types of cleft. Since nasal turbulence is usually due to a small gap,^20^ its higher prevalence in children with SPO is not surprising. Nonetheless the lack of a significant relationship between cleft type and speech outcomes probably implies that, between the years 2011 and 2015, there were other influential factors (like surgical techniques, surgeon’s experience and efficiency of the provided care) which weaken speech outcomes in almost all types of cleft palate in Iran.

There were some limitations in the current study which need to be acknowledged. Many of the children studied here had received primary surgical management at other treatment centers. Therefore, different surgical procedures may have been applied by different surgeons. Surgical technique and surgeon’s experience have been reported as very important factors that can affect palatal function and consequently speech outcomes.^21^, ^22^ Moreover, the percentage of children who had developed an oronasal fistula after cleft repair was not evaluated in our study. Furthermore, although we excluded children with identified genetic syndromes associated with cleft palate, there may be patients with unidentified syndromes in our population. Also, despite the exclusion of children with sensorineural hearing loss, a considerable percentage of our patients showed a history of middle ear and hearing issues that could have effects on their speech and language development. Speech outcomes in children with cleft palate may be influenced by all of these factors and many others. Therefore, they can also affect the relationship between speech outcomes and cleft type/age of palatoplasty. In addition, we did not access the results of functional exams (like nasoendoscopy, fluoroscopy, etc.) in this study. Visualizing soft palate and pharyngeal walls movement could help to evaluate the outcomes of palatal closure more accurately.^23^

## Conclusion

Our findings indicated the high prevalence of different speech disorders in preschool children with repaired cleft palate who visited for routine examination in ICCT from 2011 to 2015. Further, age at the time of palatal surgery was significantly associated with significant hypernasality and compensatory misarticulations. Compared with other studies, however, the prevalence of different speech disorders was also high between those children who received palatal surgery before 13 months. The age at palatal surgery, therefore, cannot be the only reason for the observed high prevalence of complications in our cleft palate children. Team care services for cleft lip/palate are provided from 2005 in Iran and despite many advances in management programs, there are still many children who do not access the interdisciplinary team cares in their early childhood. Not all of the children studied here had received team management services. We should, therefore, try to increase accessibility of appropriate and timely management services to all children with cleft lip/palate in the country. In order to improve our services, also, future studies should be carried out to examine the efficacy of the provided care.

## Conflicts of interest

The authors declare no conflicts of interest.
